# Role of Pre-operative Correction of Vitamin D3 Deficiency in Controlling Post-operative Bone Pain after Unicompartmental Knee Arthroplasty

**DOI:** 10.5704/MOJ.2411.002

**Published:** 2024-11

**Authors:** AM Rajani, ARS Mittal, VU Kulkarni, KA Rajani, KA Rajani

**Affiliations:** 1 Department of Orthopaedics, Orthopaedic Arthroscopy Knee and Shoulder Clinic, Mumbai, India; 2 Department of Clinical Research, Orthopaedic Arthroscopy Knee and Shoulder Clinic, Mumbai, India

**Keywords:** unicompartmental knee arthroplasty, vitamin D deficiency, vitamin D_3_, bone pain, shin tenderness

## Abstract

**Introduction:**

Hypovitaminosis D plays an important role in post-operative bone pain and muscle strength in arthroplasty surgeries. Its role in unicompartmental knee arthroplasty (UKA) has not been elucidated yet. The objective of this study was to determine the impact of hypovitaminosis D and its correction on post-operative bone pain after UKA.

**Materials and methods:**

A prospective cohort study involving 240 patients undergoing mobile-bearing medial UKA was conducted. Group A (na=80) received postoperative correction of Vitamin D3 Deficiency (VDD), Group B (nb=80) received pre-operative correction of VDD, while Group C (nc=80) had normal Vitamin D3 levels to begin with (≥30ng/ml). Correction was done by three doses of intramuscular injection of 600,000 IU Arachitol® (Vitamin D3) given at an interval of one week each. All groups were matched for demography and outcome measures. The level of bone pain by checking for tibial shin tenderness quantified by the visual analog scale (VAS) and evaluated pre-operatively, and at 2, 4, 6 and 12 weeks post-operatively.

**Results:**

Group B and C showed similar post-operative trends and remained significantly superior to Group A till the 6th-week follow-up. The biostatistical difference between Group A and the other two groups started decreasing after the completion of post-operative correction regime as noticed on the 6th-week follow-up. By 12 weeks post-operatively, all three groups had similar levels of bone pain.

**Conclusion:**

Vitamin D3 serves as an important preoperative investigation in patients undergoing UKA as it is a modifiable risk factor affecting post-operative bone pain. Its correction pre-operatively gives excellent post-operative pain control.

## Introduction

Vitamin D3 is a secosteroid produced in the skin in response to sunlight and regulates calcium and phosphate levels^1^. Levels of 25-Hydroxy Vitamin D3 below 30ng/ml (<72.5nmol/L) are generally considered insufficient and below 20ng/mL (<50nmol/L) considered deficient^2-3^. Insufficiency is common due to limited sun exposure, poor dietary intake, rapid urbanisation, and high air pollution or as a consequence of gut malabsorption or failure to metabolise Vitamin D3 to its active form^4^. Apart from its well-known role in the modulation of osseous and serum calcium and phosphate levels, Vitamin D3 has an important role in perception of pain via the modulation of anti-inflammatory cytokines^5^. The prevalence of VDD has been reported to be as high as 59% in literature, with an increasing trend in the older age group^6^. This is of significance in patients undergoing surgery for degenerative conditions such as osteoarthritis of the knee joint wherein an 82% prevalence has been reported^7^. Although the causal role of low Vitamin D3 on pain due to osteomalacia is well recognised, the combination of surgery and hypovitaminosis D may be further deleterious^8^. Studies have shown low or insufficient levels of Serum Vitamin D3 resulting in suboptimal recovery in patients undergoing total knee arthroplasty (TKA)^9^. Preoperative Serum Vitamin D3 insufficiency was also proposed as a modifiable risk factor for moderate to severe, persistent pain after TKA in addition to poorer quality of life8,10. Hypovitaminosis D is also related to worse pain-related outcomes and quality of life^10^. Peri prosthetic infection and longer hospital stay have also been reported with VDD^11^. Apart from enzyme linked immunosorbent assay (ELISA) as a chemical method to measure Vitamin D3 levels, tibial bone tenderness can be used as a clinical sign for VDD^12^. Over the past decades, orthopaedic surgeons have made a constant effort to ameliorate bone pain after arthroplasty surgeries. Considering the rapidly increasing number of UKA procedures being done across the globe owing to its superior results to TKA in patients with bone-on-bone medial compartment osteoarthritis (MCOA)^13^, the influence of VDD in such patients could be important.

The effect of VDD on post-operative bone pain after a UKA is still unknown, hence this study attempted to fill this lacuna by assessing the role of pre-operative VDD correction on post-operative bone pain by assessing the tibial shin tenderness and quantifying it using VAS scores in patients undergoing UKA. We hypothesised that pre-operative correction of VDD in such patients gives superior results in terms of bone pain control as compared to patients who underwent correction after the surgery and in the bigger picture, help in improving overall or recalcitrant pain. This will consequently help in establishing whether or not Vitamin D_3_ is an important pre-operative investigation protocol for UKA in order to obtain better results.

## Materials and Methods

This was a prospective cohort study including 240 consecutives, consenting patients who underwent primary UKA between 1st January 2020 and 31st March 2022, performed by a single surgeon at a single centre. All procedures that were followed were in accordance with the ethical standards of the Helsinki Declaration of 1975, as revised in 1983. Informed written consent was taken from all the patients before including them in the study. The patients were recruited based on fixed inclusion and exclusion criteria. Patients clinically and radiologically diagnosed medial compartment osteoarthritis were counselled for the study and advised magnetic resonance imaging (MRI) (n=712). Patients with more than 10° of varus or 5° of valgus deformity, flexion contracture, or unwilling to participate in the study were excluded (ne=211). Patients diagnosed with lateral compartment osteoarthritis on MRI were advised total knee arthroplasty and excluded from study (ne=73). Further exclusions were patients undergoing revision surgery, patients on medications (antiepileptics, corticosteroids, immunosuppressive agents, chemotherapeutic agents, thiazides) or conditions (advanced kidney disease (eGFR <60mL/minute) or on dialysis support, hypercalcemia (total calcium >10.6mg/dL or ionized serum calcium >5.4mg/dL), nephrolithiasis, hyperparathyroidism, obesity/body mass index (BMI) of more than 30kg/m^2^) affecting vitamin D3 absorption and metabolism, patients already on Vitamin D3 supplements, patients having a known contraindication to Vitamin D_3_ supplements such as hypersensitivity, or conditions that could confound the outcome of surgery such as granulomatous disease, previous infection/septic arthritis in the same joint, uncontrolled diabetes mellitus (HBA1c>8%), patients with neuromuscular disorders or prolapsed intervertebral disc (ne=177).

**Fig. 1:**
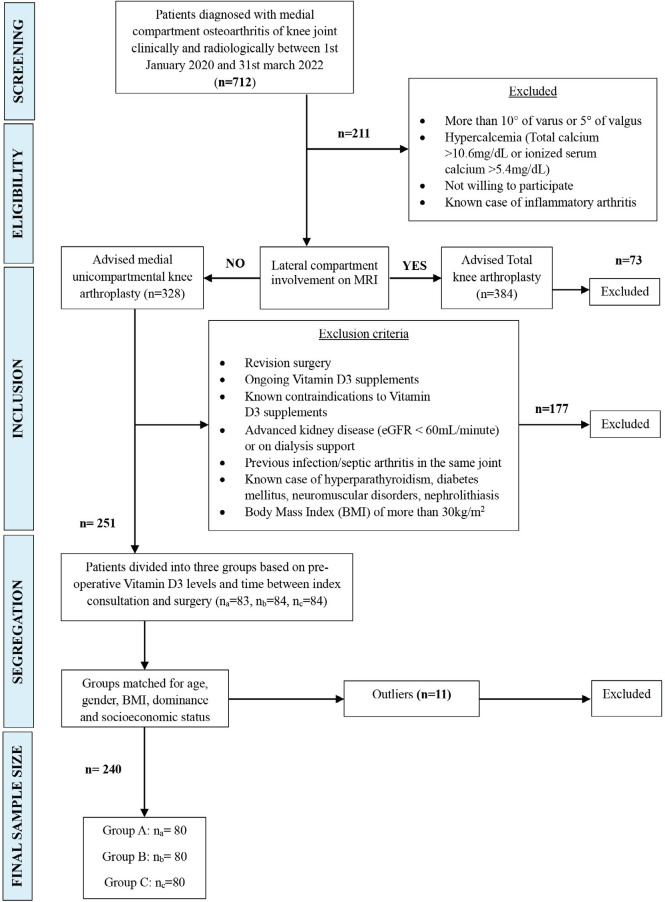
PRISM chart demonstrating recruitment protocol of the sample size and segregation into individual groups.

**Fig. 2:**
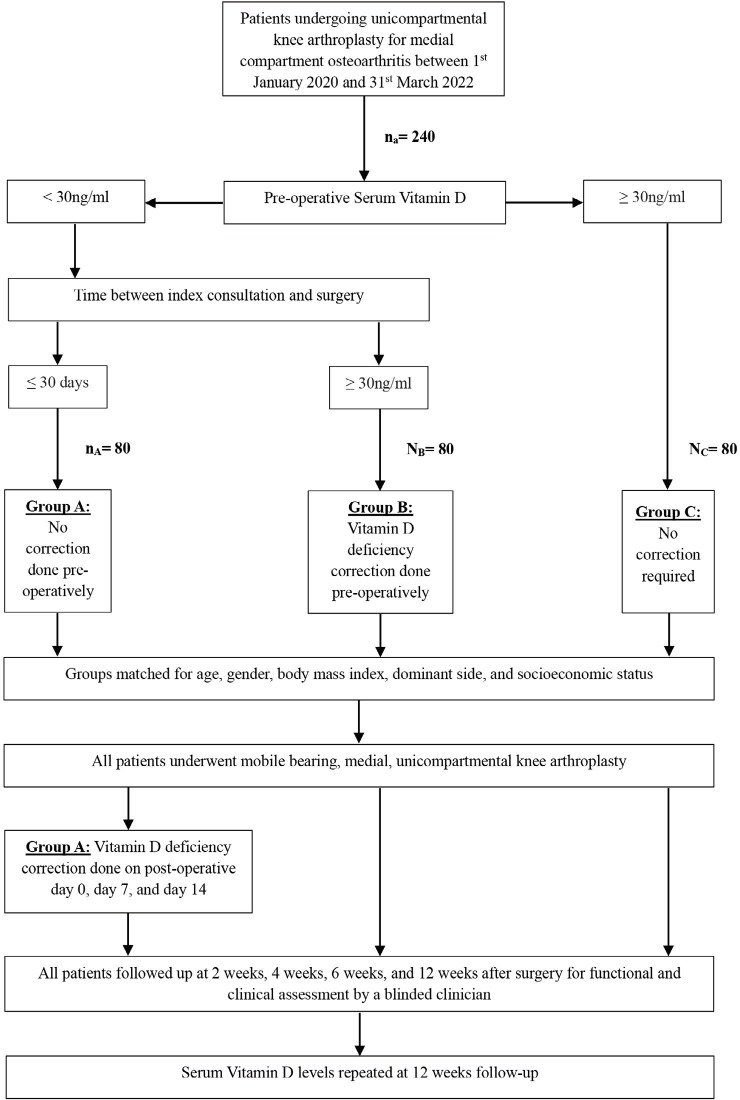
Flowchart representation of selection process of sample size, intervention process, and outcome assessment.

All the patients enrolled in the study were diagnosed as a case of MCOA knee by clinical examination and radiologically using standard anteroposterior standing radiographs, lateral supine radiographs, and special radiographs such as Rosenberg posteroanterior view and Valgus stress anteroposterior view for correctability of varus deformity. The demographic data of all the patients was collected and compiled. All these patients were also evaluated pre-operatively for bone pain by clinically assessing VDD by assessing for tibial shin tenderness. Visual analog scale (VAS) was used for pain evaluation^14,15^. Preoperative levels of Serum Vitamin D_3_ were calculated using the Electrohemiluminescence Immunoassay method from the same diagnostic centre (blinded) and samples collected early morning after overnight fasting. The cut-off for VDD in our study was kept at 30ng/ml^3^. Patients with VDD who were scheduled for surgery ≤30 days from their index consultation underwent post-operative correction of Vitamin D_3_ levels and were allocated Group A (na=80). Those scheduled for surgery >30 days from their index consultation underwent pre-operative correction of the VDD and were classified as Group B (nb=80). Group C (nc=80) included patients with normal Vitamin D_3_ levels from the outset on pre-operative testing and operated within a month of evaluation. The three groups were matched for age, gender, BMI, dominant side, comorbidities (including medications), and socioeconomic status (according to the modified Kuppuswamy scale)^16^. Correction was done by three doses of injection Arachitol^®^ 600,000 IU (Abbott Healthcare Products BV) given intramuscularly (IM) once a week for three weeks based on the experience of the chief surgeon (Fig. 1, Fig. 2).

All the 240 patients enrolled in the study underwent UKA by mobile bearing Oxford knee arthroplasty by the same surgeon, at the same centre, under spinal anaesthesia. Paramedian incision and mini mid-vastus approach^17^ was used and all patients had a similar incision size of about 8cm with minimal soft tissue insult and use of cautery. All the individuals in group A and group B had post-operative bone pain assessed by severity of shin tenderness which was attributed to deficient serum Vitamin D_3_ levels despite of the fact that all the individuals enrolled in this study were given the same analgesics and anti-inflammatory medications postoperatively for a period of one week only. Similar preoperative, intra-operative, and post-operative protocols were followed strictly. On the day of the surgery, intravenous paracetamol 1gm was given at 8-hour interval to all patients. From day one post-surgery, patients were started on the oral etoricoxib regimen (60mg TDS) till post-operative day seven. All patients underwent the same rehabilitation protocol with mobilisation using walker from the day of the surgery. Correction of VDD in Group A was done postoperatively starting from the night after the surgery.

Post-operative assessment was done by a blinded clinician at 2,4,6 and 12 weeks after the surgery by documenting the VAS score for shin tenderness (Fig. 2). No patient was lost to follow-up. All of the data was compiled and computed on the SPSS software, version 21; SPSS Inc., [Chicago, IL, USA]. Longitudinal intragroup assessment of individual groups for improvement in VAS score for shin tenderness was done using repeated measure ANOVA test and pairwise comparison. The Kruskal-Wallis test was applied to compare the bone pain of each group with one another (Group A vs Group B vs Group C) at every follow-up. A power analysis of the study for a total sample size of 240 revealed a power score of 100% when keeping ∝ as 0.05, and degree of freedom (df) as 2. Analysis for the sample size of each group as 80, keeping VAS score as the dependent variable, adjusted R squared as 0.340, ∝ as 0.05, and df as 2 also revealed a power score of 100%.

## Results

A total of 240 patients (n=240) were included in the study after matching, out of which 154 (64.17%) were females and 86 (35.83%) were males. The demographic data of the sample size was as elucidated in Table I. The mean age of the sample size was 64.8±8.29 years. The mean Vitamin D_3_ levels of Group A at index consultation was 16.8±6.08ng/ml while that of Group B was 16.27±6.76ng/ml. The mean Vitamin D_3_ levels of Group B after correction with three intramuscular injections of 600,000 IU Arachitol^®^ in the preoperative period was 51.02±6.45ng/ml.

**Table I:** Baseline demographic and clinical data of patients in Group A (Corrected after surgery), Group B (Corrected before surgery) and Group C (No Vitamin D deficiency).

Variables	Group A	Group B	Group C	Overall
Number of patients (n)	80	80	80	240
Mean Age (years)	64.34 ± 8.54	65.46 ± 8.20	64.62 ± 8.20	64.8 ± 8.29
Gender				
Males	29 (36.3%)	28 (35%)	29 (36.3%)	86 (35.83%)
Females	51 (63.7%)	52 (65%)	51 (63.7%)	154 (64.17%)
Side affected				
Dominant	41 (51.25%)	40 (50%)	42 (52.5%)	123 (51.25%)
Non-dominant	39 (48.75%)	40 (50%)	38 (47.5%)	117 (48.75%)
Mean Body Mass Index (kg/m2)	27.24±2.38	26.8±2.97	27.15±2.04	27.06±2.48
Pre-operative Vitamin D (ng/ml)	16.8±6.08	51.02±6.45	48.6±7.32	38.81±6.62
12 weeks follow-up Vitamin D (ng/ml)	52.37±6.74	50.48±6	47.95±7.18	50.26±19.92
Comorbidities (on medication)				
Hypertension	24 (30%)	22 (27.5%)	22 (27.5%)	68 (28.33%)
Diabetes Melltitus (HBA1c<8%)	4 (5%)	6 (7.5%)	3 (3.75%)	13 (5.42%)
Hypothyroidism	2 (2.5%)	1 (1.25%)	2 (2.5%)	5 (2.08%)
Ischemic Heart disease	2 (2.5%)	3 (3.75%)	3 (3.75%)	8 (3.33%)
Anxiety/ Depression	1 (1.25%)	1 (1.25%)	1 (1.25%)	3 (1.25%)
Socioeconomic status (Modified Kuppuswamy scale)				
Upper	17 (21.25%)	15 (18.75%)	16 (20%)	58 (24.17%)
Upper middle	16 (20%)	16 (20%)	18 (22.5%)	50 (20.83%)
Lower middle	24 (30%)	27 (33.75%)	26 (32.5%)	67 (27.92%)
Upper Lower	23 (28.75%)	22 (27.5%)	20 (25%)	65 (27.08%)
Lower	0 (0%)	0 (0%)	0 (0%)	0

Notes: All values presented as mean ± standard deviation (95% confidence interval) or number (percentage)

The VAS score for shin tenderness at second-week postoperative follow-up was insignificantly less as compared to the pre-operative VAS (p>0.05). The VAS score at 4 weeks, 6 weeks, and 12 weeks, however, was significantly better than the preceding follow-up and the pre-operative VAS (p<0.05) in all three groups (Table II).

**Table II:** Intergroup comparison of bone pain by quantifying shin tenderness in terms of visual analogue score between Group A (correction after surgery) and Group B (correction before surgery) across all follow-ups using Kruskal-Wallis test. Visual Analog Scale for severity of shin tenderness.

Follow-up duration	Visual Analog Scale for severity of shin tenderness		
	Group A (na = 80)	Group B (nb = 80)	p-value
Pre-operative	7.06 ± 1.12	5.28 ± 1.32	<0.0001
2 weeks	6.85 ± 1.06	5.11 ± 1.26	<0.0001
4 weeks	5.19 ± 1.21	3.13 ± 1.42	<0.0001
6 weeks	3.68 ± 1.04	1.06 ± 0.96	<0.0001
12 weeks	0.35 ± 0.62	0.33 ± 0.5	0.597

Notes: All values presented as mean ± standard deviation (95% confidence interval). P-value <0.05 is considered significant (bold).

**Fig. 3:**
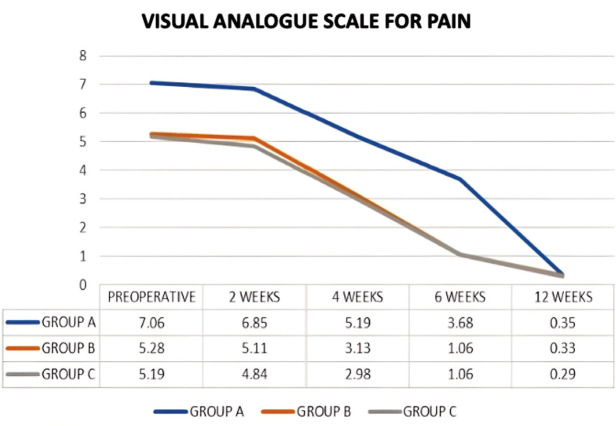
Line diagram charting temporal trends of the three groups over the course of the follow-ups to compare the Mean Visual Analogue score for severity of shin tenderness.

On intergroup comparison it was noted that Group B had similar VAS scores as compared to Group C throughout the follow-up period. Group B which underwent VDD correction prior to surgery had a significantly better VAS score (p<0.001) at the immediate pre-operative period. On post-operative follow-up at week two, week four and week six, Group B had statistically superior VAS score compared to Group A which underwent VDD correction after surgery (Table II). However, there was an improving trend in mean scores of Groups B as compared to Group A once correction of VDD was completed (Fig. 3). By the 12th week follow-up, both the groups that underwent VDD correction before and after surgery had comparable VAS score for shin tenderness (p=0.597). Mean serum Vitamin D levels repeated at 12 weeks follow-up showed similar values in both groups (p=0.14). Group B had similar VAS score as compared to Group C throughout the follow-up period.

## Discussion

Our study demonstrated that pre-operative diagnosis and correction of VDD (Vitamin D_3_ <30ng/ml) by three, weekly, intramuscular injections of Arachitol^®^ 600,000 units each, in patients planned for UKA gives excellent post-operative results. Role of supplementation of Vitamin D_3_ deficiency prior to TKA surgery and its trends has been reported across literature, with no description of its influence on those undergoing UKA. The nutritional status of Vitamin D_3_ is best reflected by the main circulating metabolite 25-Hydroxy Vitamin D_3_ (25(OH)D_3_). Low levels of Vitamin D_3_ have been reported in 22-36% of patients with Hip osteoarthritis by Bischoff-Ferrari *et al*^18^ and up to 66% in patients with Knee osteoarthritis by Glowacki *et al*, as was the case in our study too^19^.

Type 2 muscle fibres have a greater amount of VDR’s than type 1 muscle fibres therefore supplementation in deficient patients leads to the proliferation of type 2 muscle fibres^20^, consequently resulting in muscular development and function by regulating phosphate accumulation in the myocytes thereby aiding in muscle function as well^21^. This presumably explains improved bone pain and muscular strength after correction of VDD. Vitamin D_3_ levels are directly linked to the articular cartilage thickness as well. It prevents cartilage loss through regulation of type 2 collagen, hence VDD has a deleterious effect on the articular cartilage thereby accelerating the progression of osteoarthritis^22^.

The demographic distribution of our sample size was similar to previous studies involving patients with MCOA of the knee joint as documented by Feng *et al* in 2019^23^. The prevalence of VDD in our sample size was 66% which was similar to the numbers reported by Song *et al*^24^. Injectable (IM) route of Vitamin D_3_ supplementation was chosen in our study due to evidence of its efficacy, rapidity, and longevity of correction as opposed to oral supplementation which may take upto three months for correction^18,25^. Supplementation of VDD was done with 600,000 IU of IM Arachitol^®^ injections given at one-week intervals for three weeks, based on the experience of the authors. Toxicity was ruled out by looking for concerned signs and symptoms at every follow-up^26^ and repeating the serum Vitamin D_3_ levels at 12-week follow-up. Cut-off for hypervitaminosis D was 100ng/ml and toxicity was 150ng/ml as per the guidelines laid down by the Endocrine society and elucidated by Vogiatzi *et al* in 20143. The mean Vitamin D_3_ levels of the three groups remained well within therapeutic range on 12 weeks follow-up (Group A: 52.37±6.74; Group B: 50.48±6; Group C: 47.95±7.18).

Barker *et al*^27^ in their study reported deleterious effects of surgery in patients with insufficient Vitamin D_3_ levels, while Maniar *et al* in 2016^9^ assessed the negative impact of VDD on functional outcomes after a TKA. Similarly, we noted that supplementation of Vitamin D_3_ pre-operatively significantly modulated post-operative bone pain by observing a better VAS score of shin tenderness in patients who underwent correction pre-operatively, which was similar to the group that had normal Vitamin D_3_ levels to begin with. The findings of Maniar *et al*^9^ was also congruent to our study with respect to improvement of function on post-operative supplementation of Vitamin D_3_, however, unlike postoperative oral supplementation done in that study, we recommend pre-operative parenteral supplementation to safely expedite the correction process and minimise patient non-compliance.

Tomlinson *et al* in his study noted a significant increase in muscle strength when supplemented with Vitamin D_3_. Patients with deficient levels of Vitamin D_3_ had higher odds of Hamstring strain compared with those with sufficient levels^28^. Similarly, we noted that supplementing the deficient groups improved the post-operative bone pain, muscle strength and exercise tolerance. Vitamin D_3_ guideline put forth by the National Osteoporosis Society states that those patients with an underlying bone disease that can potentially be improved with Vitamin D_3_ supplementation or those who need Vitamin D_3_ deficiency corrected prior to specific surgical intervention should have their Serum Vitamin D_3_ levels measured pre-operatively^29^. The impact on the postoperative functional outcome is presumably related to Serum Vitamin D_3_ levels as it modulates the inflammatory response. Hwang *et al*^30^ reported in 2020 that pre-operative Vitamin D_3_ levels does not affect short term functional outcome in elderly women after TKA. However, there is little to no evidence of the effect of the same in UKA.

This study helps in defining the role of Vitamin D3 deficiency as an important modifiable risk factor for postoperative bone pain even after a relatively bone and soft tissue conserving surgery like UKA. It advocates for routine inclusion of Serum Vitamin D_3_ levels as an important preoperative investigation before a UKA and preferably correction of any VDD before the surgery to give better postoperative results. The three groups were matched for age, gender, BMI, and dominance, thereby minimising any confounding effect in data collection. Other confounding factors to pain that were assessed included pre-operative demographic characteristics, patellofemoral involvement, deformity, psychosocial factors, deformity correction, impingement, infection, referred pain, malalignment, instability stiffness, impingement, implant loosening, speed and duration of mobilisation and rehabilitation. The clinicians as well as the diagnostic centres were blinded to patient data, hence eliminating bias. The authors acknowledge some limitations to the study. This study was done to evaluate the short-term effects of VDD in the immediate post-operative period and warrants for more research on the long-term effects of such corrections. The geographical area for the collection of the sample size was restricted and studies are required with a more diverse population pool to eliminate genetic and racial bias. A placebo or untreated group was not used for comparison and more studies incorporating the same could enhance external validity of the study. All the patients underwent only mobile bearing Oxford UKA, hence its effect on fixed bearing UKA cannot be extrapolated. All the patients were examined by a single, blinded clinician, thereby leaving a scope for human error. The study was conducted by a single surgeon and at a single centre, limiting the generalisability of the findings, hence we encourage more multicentric studies, with a more diverse, and larger sample size to validate our results. Finally, as all the procedures were done by a single surgeon, the reproducibility needs to be fortified by similar studies.

## Conclusion

With the trend shifting to unicompartmental knee arthroplasties in the patients with medial compartment osteoarthritis, the prevalence of Hypovitaminosis D seen in these individuals is also on a rise. We propose timely diagnosis of hypovitaminosis D (Serum Vitamin D_3_ levels <30ng/ml) by including serum Vitamin D_3_ levels as a routine pre-operative investigation and its requisite correction by administration of 600,000 IU of Arachitol^®^ IM (three doses at an interval of one week each) in deficient patients pre-operatively for excellent post-operative control of bone pain and by extension, overall control of acute and recalcitrant pain in patients.
